# The VEPRO trial: A cross-over randomised controlled trial comparing 2 progressive lenses for patients with presbyopia

**DOI:** 10.1186/1745-6215-9-54

**Published:** 2008-09-19

**Authors:** Isabelle Boutron, Caroline Touizer, Isabelle Pitrou, Carine Roy, Philippe Ravaud

**Affiliations:** 1 INSERM, U738, Paris, France; 2 Université Paris, 7 Denis Diderot, UFR de Médecine, Paris, France; 3 AP-HP, Hôpital Bichat, Département d'Epidémiologie, Biostatistique et Recherche Clinique, Paris, France; 4 Santéclair, Boulogne, France

## Abstract

**Background:**

The aim of this trial was to compare the effectiveness of two generations of progressive lenses for presbyopia.

**Methods:**

A multicenter cross-over randomized controlled trial performed in a primary care setting (5 optical dispensaries) was planned. Two categories of progressive lenses were compared: 1) a new-generation lens (i.e., VARILUX PANAMIC ORMA CRIZAL), which is expensive but a supposed improvement in comfort, and 2) an older-generation lens (i.e., VARILUX CONFORT ORMA CRIZAL), which is less expensive and is considered the reference lens. Patients were randomized to wear one generation of progressive lens for 4 weeks, then cross over to wear the other lens for 4 weeks, without knowing the sequence of lenses.

Inclusion criteria were 1) age 43–60 years; 2) outpatients already wearing progressive lenses and referred to an optician ophthalmologist for optical correction prescription within the last 6 months; 3) receiving a correction of ≤3 dioptres in cases of associated myopia, hyperopia or astigmatism; 4) understanding and speaking French and able to answer a questionnaire; and 5) giving written consent to participate in the study.

The primary outcome was patient preference for one progressive lens at week 8. Secondary outcomes were subjective measures of bifocal visual performance, including a) near visual acuity, b) visual field, c) kinetic visual skills, d) visual adaptability, e) visual comfort, and f) rapidity of adaptation.

**Results:**

127 patients were randomized to one of the lens groups. Two patients withdrew prematurely; 98.4% and 97.6% patients who wore the new versus older lenses, respectively, wore their progressive lenses every day during the 4-week period 1 and period 2. The number of participants in each of 5 centres varied from 16 (12.6%) to 35 (27.6%).

57.9% patients preferred the new-generation lenses, 36.5% the older-generation lenses, and 5.6% had no preference (p = 0.01). The two groups did not differ in any of the measures of bifocal visual performance except near visual acuity.

**Conclusion:**

Patients with presbyopia had slightly higher preference for the new-generation than older-generation lens, with no difference in lens groups for most of the visual outcomes assessed.

**Trial Registration:**

ClinicalTrials.gov NCT00635115

## Background

Presbyopia is a chronic disorder involving changes in accommodation of the eye and causing difficulty in near vision. It is a process of age-related progressive loss of accommodative amplitude [1,2]. Presbyopia is associated with worse health-related quality of life in terms of vision [3-5]. Correction of presbyopia includes bifocal or trifocal lenses, progressive lenses, bifocal or progressive contact lenses and surgery [6,7]. Because of continuous improvement in design of progressive lenses, these have become the treatment of choice for many practitioners [8,9]. Since the first progressive lenses, several types of progressive lenses with varying costs have been proposed. Essilor is the French leader in manufacturing progressive lenses and has developed a succession of lenses, including VARILUX CONFORT and VARILUX PANAMIC. Although VARILUX PANAMIC is more expensive, its effectiveness has never rigorously been compared to the older-generation VARILUX CONFORT.

We aimed to perform a randomized controlled trial comparing the effectiveness of two progressive lenses for presbyopia – a new-generation lens (VARILUX PANAMIC ORMA CRIZAL) and an older-generation progressive lens (VARILUX CONFORT ORMA CRIZAL)-in terms of patient preference and subjective measures of bifocal visual performance.

## Methods

### Design

A multicenter cross-over randomized controlled trial was designed. This design removes patient variation and allowed us to estimate treatment effect with greater precision: individual response to treatment A was compared with the same subject's response to treatment B. This design was particularly fitting because presbyopia is a chronic disease with short-term outcomes, and the treatment does not interact with the underlying disorder. Furthermore, patients included were already wearing progressive lenses for presbyopia. They were referred to an optical dispensary with an optical correction prescription. Consequently, the carry-over effect is probably negligible [10].

The trial was approved by the institutional review board of Cochin Hospital, Paris. Informed consent was obtained from all participants, and all clinical investigations were conducted according to the principles of the Declaration of Helsinki. This study report followed the guidelines of the CONSORT Statement and its extension to reports of nonpharmacological treatments [11-13].

### Participants

Optical dispensaries participating in this trial were selected from partners of the healthcare insurance companies Santéclair and Groupama in France. Both companies were involved in the funding of the trial. Five optical dispensaries based in Paris or nearby agreed to participate. They were committed to inform patients, check selection criteria, obtain informed consent, include patients, administer the allocated progressive lenses, and collect information.

To be included in the trial, patients had to be 1) 43 to 60 years old; 2) outpatients already wearing progressive lenses for presbyopia and consulting an optical dispensary with an optical correction prescription within the last 6 months; 3) receiving correction of = 3 dioptres in cases of associated myopia, hyperopia or astigmatism; 4) understanding and speaking French and able to answer a questionnaire; and 5) giving a written consent to participate with the study. Exclusion criteria were 1) first prescription of progressive lenses for presbyopia, 2) associated strabism, 3) associated amblyopia, 4) receiving orthoptics therapy, 5) associated anisometropia > 1.5 dioptres, and 6) receiving treatment for diabetes.

Patient screening was organized by the healthcare insurance company Santéclair which has a consumer panel of specifically trained counsellors. Consumers could contact the panel for advice and to be referred to one of the optical dispensary partners of the insurance companies. All patients contacting the panel and meeting inclusion criteria were invited to participate. They were referred for inclusion if they agreed to use a participating optical dispensary. A standardized text was used to check patient eligibility. The text is available upon request.

### Sequence allocation generation

Patients were randomly assigned to 2 treatment sequences: 1) use of the older-generation progressive lens (i.e., VARILUX CONFORT ORMA CRIZAL) for 4 weeks followed by the new-generation lens (i.e., VARILUX PANAMIC ORMA CRIZAL) for 4 weeks; or 2) use of the new-generation lens (i.e., VARILUX PANAMIC ORMA CRIZAL) for 4 weeks followed by the older-generation lens (i.e., VARILUX CONFORT ORMA CRIZAL) for 4 weeks. Randomisation was stratified on optical dispensaries. A randomisation code was generated by a statistician at the Department of Epidemiology, Biostatistics and Clinical Research, Bichat Hospital, who was not involved in the conduct and analysis of the study. A computer random-number generator was used to select random permuted blocks, with block lengths of 4, 8, and 10.

### Allocation concealment

To ensure allocation concealment, the random list was sent to an independent central laboratory in charge of assembling all the equipment for 5 optical dispensaries. The list was not provided to the optical dispensaries or to any sponsors. After obtaining informed consent from the patient, the optical dispensary asked for a patient's inclusion number by faxing an inclusion form to Santéclair. For the purpose of the study, Santéclair was also in charge of allocating patients' inclusion numbers by order of fax arrival. With this patient's inclusion number, the optical dispensary ordered from the central laboratory two sets of equipment for patients. A set of equipment consisted of one pair of progressive lenses and one spectacle frame. Then the central laboratory sent the equipment to the optical dispensary and indicated with an appropriate blinded label the sequence of use of each set.

### Intervention

We compared two categories of progressive lenses: 1) the new-generation lens (i.e., VARILUX PANAMIC ORMA CRIZAL), which is expensive but a supposed improvement in comfort, and 2) an older-generation progressive lens (i.e., VARILUX CONFORT ORMA CRIZAL), which is less expensive. Both lenses were indexed 1.5 and had the same anti-reflection coating treatment. These properties were chosen according to usual optical corrections proposed to patients. Patients had to choose among a limited range of spectacle frames (10 styles). When participating in the trial, patients did not have to pay for the spectacle frame and the progressive lenses. Patients were allowed to keep the preferred equipment at the end of the trial as in incentive for taking part in the trial.

### Blinding

In this trial, the primary and secondary outcomes were patient-reported outcomes. Because outcomes were subjective and the outcome assessor was the patient, blinding of the patients was particularly important. To ensure adequate blinding, the equipment provided to the patients was strictly similar: same spectacle frame, colour, shape, and weight. The two sets of equipment were assembled in the central lab on the same day by the same technician. The only variable to be tested was therefore the generation of the progressive lenses. All progressive lenses were provided by the same manufacturer (i.e., Essilor). However, all lenses manufactured by Essilor are engraved with a tiny mark, so people aware of the mark could guess the assigned lens. To ensure the success of blinding, patients were not informed of this mark, and opticians were committed to not checking the treatment administered and not informing patients of the distinction.

### Outcomes

To assess outcomes, patients were clinically evaluated by their opticians during visits at baseline and at weeks 4 and 8. The primary outcome was evaluated at the final visit (i.e., week 8), and secondary outcomes were assessed during the follow-up period (i.e., weeks 4 and 8). The primary outcome was patient preference for a progressive lens based on period of wear. Patients had to indicate the period they preferred on a scale of -5 to 5 (i.e., -5 to -1: preference for period 1; 0: no preference; 1 to 5: preference for period 2). Secondary outcomes were subjective measures of various areas of bifocal visual performance. These were measured on a scale of 0–10 (i.e., 0, extremely bad; 10, excellent) and included assessment of a) near visual acuity (i.e., < 40 cm: read a book), b) distance visual acuity (i.e., > 5 m: watch a movie in a cinema, look at an advertising slot or a road sign), c) intermediate visual acuity (i.e., 40 cm to 5 m: computer work, look at prices through a shop window or people around a table), d) global visual acuity, e) distance visual field (i.e., look at the other side of the road and focus on the sharp area around something you stare at), f) near visual field (i.e., stare at a letter in a book and evaluate the sharp area around this letter), g) kinetic visual skills when the person is moving but the environment is still (i.e., walk and stare at prices through a shop window); h) kinetic visual skills when the person is still but the environment is moving (i.e., driving), i) visual adaptability, and j) visual comfort. Finally, we assessed rapidity of visual adaptation to progressive lenses on an 8-point Likert scale (immediately, in few hours, 1 day, 2–3 days, 1 week, 2 weeks, more than 2 weeks, never). We considered that visual adaptation was faster when reported as immediate or in few hours.

### Adverse events

All adverse events were systematically collected. We found no expected adverse events. Severity was classified according to the World Health Organization classification. All severe adverse events were systematically reported by the optical dispensaries. The relation between intervention and adverse events was assessed by the optician.

### Sample size

The primary outcome was patient preference for a progressive lens based on period of wear: new-generation or older-generation lens or no preference. We calculated sample size on the basis of the hypothesis that 40% patients would prefer the new-generation lens, 40% would have no preference and 20% would prefer the older-generation lens. If we consider only patients expressing a preference, we would compare patients preferring the new-generation lens (i.e., 66.7% = 40/(40+20)) to a theoretical 50%. With an alpha risk of 5% and a power of 80%, the estimated sample size was 68 patients. Considering that about 40% patients would not provide informative data (i.e., would have no preference) and a rate of lost to follow-up of about 15%, we aimed for a sample of 130 patients.

### Statistical methods

Statistical analysis was performed by a blinded statistician (CR) at the Department of Epidemiology, Biostatistics and Clinical Research, Bichat Hospital. Analyses were conducted according to a pre-specified plan based on the principle of intent-to-treat (i.e., data for all participants were analysed in the group participants were assigned to, regardless of whether they completed the intervention). Descriptive statistics (mean and standard deviation [SD], extreme values) were used for continuous variables. Categorical variables were described with frequencies and percentages. The primary outcome was evaluated by a 2-tailed Prescott's test (i.e., takes into account the effect of period and patients with no preference). Secondary outcomes were evaluated by an F test: a linear mixed-effects model, with the secondary outcome as the dependent variable and fixed effects for sequence, study group, and period, with patients within sequence as a random effect. Data analyses involved use of SAS 9.1 (SAS Institute Inc, Cary, NC, USA).

## Results

### Participants

**Figure 1:**
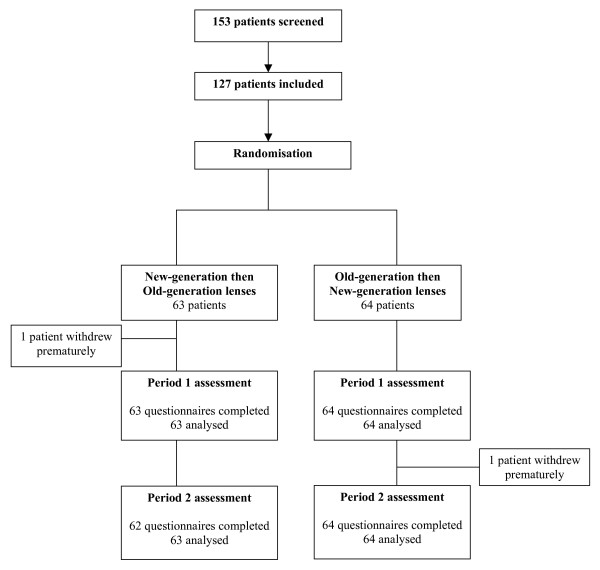
Flow diagram of patients through the trial.

Patients were screened and enrolled from February to November 2006. The flow of participants through the trial is reported in figure 1. Baseline characteristics of patients are summarized in table 1. The number of participants in each of the 5 centres varied from 16 (12.6%) to 35 (27.6%). In period 1, one patient allocated to the new-generation lens first (i.e., VARILUX PANAMIC ORMA CRIZAL) withdrew prematurely, and 98.4% patients wore their progressive lenses every day during the 4 weeks. In period 2, one patient allocated to the older-generation lens first (i.e., VARILUX CONFORT ORMA CRIZAL) withdrew prematurely, and 97.6% patients wore their progressive lenses every day during the 4 weeks.

**Table 1:** Patient characteristics at baseline

	**Total** **N = 127**	**New-generation ** **lens first** **N = 63**	**Older-generation ** **lens first** **N = 64**
**Patient characteristics**			
-Sex (male/female) [n (%)]	49/78 (38.6/61.4)	21/42 (33.3/66.7)	28/36 (43.7/56.3)
-Age (years) [mean (SD)]	53.0 (5.1)	52.1 (4.9)	53.9 (5.1)
-Time since the first prescription for progressive lens for presbyopia (years) [mean (SD)]	5.7 (4.5)	5.5 (4.8)	6.0 (4.1)
-Time since patients wore their equipment before having a new prescription for progressive lens for presbyopia (years) [mean (SD)]	2.3 (2.0)	2.1 (1.9)	2.5 (2.0)
**Reason for prescription**			
-Correction modification [n (%)]	90 (70.9)	39 (61.9)	51 (79.7)
-Lack of comfort [n (%)]	22 (17.3)	12 (19.0)	10 (15.6)
-Aesthetics [n (%)]	16 (12.6)	9 (14.3)	7 (10.9)
**Visual defect**			
-Myopia [n (%)]	51 (40.5)	27 (43.5)	24 (37.5)
-Hyperopia [n (%)]	75 (59.5)	35 (56.4)	40 (62.5)
-Astigmatism [n (%)]	89 (70.1)	46 (73.0)	43 (67.2)
**Previous correction**			
-Anti-reflection coated (yes) [n (%)]	94 (74.6)	51 (82.3)	43 (67.2)
-Sphere sign RE (+/-) [n (%)]	67/48 (58.3/41.7)	31/24 (56.4/43.6)	36/24 (60.0/40.0)
-Sphere sign LE (+/-) [n (%)]	71/47 (60.2/39.8)	33/25 (56.9/43.1)	38/22 (63.3/36.7)
-Sphere RE [mean (SD)]	1.0 (0.7)	0.9 (0.8)	1.0 (0.7)
-Sphere LE [mean (SD)]	1.0 (0.7)	1.0 (0.8)	0.9 (0.6)
-Cylinder RE [mean (SD)]	0.6 (0.3)	0.6 (0.3)	0.5 (0.3)
-Cylinder LE [mean (SD)]	0.6 (0.3)	0.6 (0.3)	0.6 (0.3)
-Axis RE [mean (SD)]	95.7 (59.0)	83.4 (57.5)	108.7 (58.5)
-Axis LE [mean (SD)]	92.3 (58.7)	99.1 (52.5)	85.3 (64.6)
-Addition RE [mean (SD)]	1.9 (0.6)	1.9 (0.7)	1.9 (0.5)
-Addition LE [mean (SD)]	1.9 (0.6)	1.9 (0.7)	1.9 (0.5)
**New correction**			
-Sphere sign RE (+/-) [n (%)]	70/50 (58.3/41.7)	30/26 (53.6/46.4)	40/24 (62.5/37.5)
-Sphere sign LE (+/-) [n (%)]	73/50 (59.3/40.7)	35/26 (57.4/42.6)	38/24 (61.3/38.7)
-Sphere RE [mean (SD)]	1.0 (0.7)	1.0 (0.8)	1.1 (0.7)
-Sphere LE [mean (SD)]	1.0 (0.8)	1.1 (0.8)	1.0 (0.7)
-Cylinder RE [mean (SD)]	0.6 (0.4)	0.6 (0.4)	0.6 (0.5)
-Cylinder LE [mean (SD)]	0.7 (0.4)	0.7 (0.4)	0.6 (0.5)
-Axis RE [mean (SD)]	101.2 (58.4)	91.7 (57.5)	112.6 (58.2)
-Axis LE [mean (SD)]	95.6 (58.6)	97.6 (53.5)	93.7 (63.7)
-Addition RE [mean (SD)]	2.1 (0.5)	2.1 (0.5)	2.2 (0.5)
-Addition LE [mean (SD)]	2.1 (0.5)	2.1 (0.5)	2.2 (0.5)

RE: Right eye, LE: Left eye

### Outcomes

**Figure 2:**
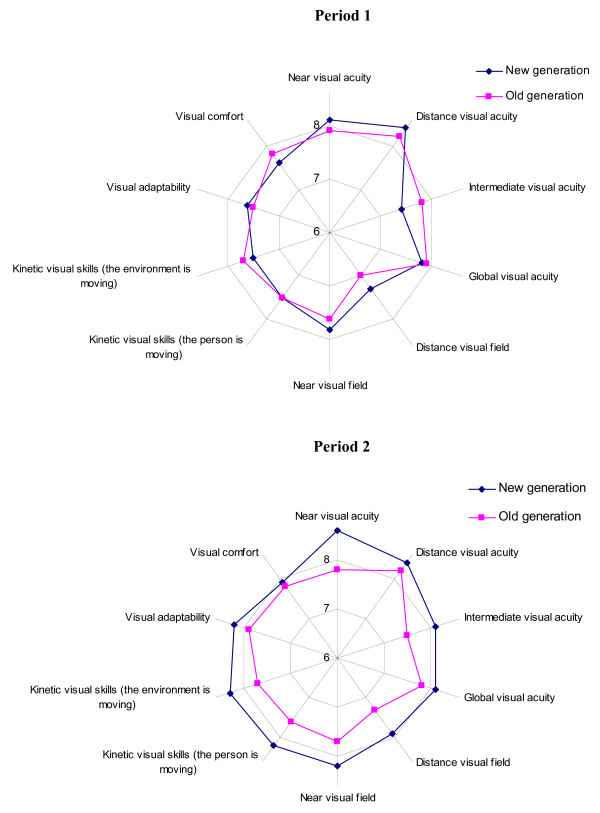
Mean scores of bifocal visual performance (0–10 numeric scale).

At the end of the follow-up, 57.9% patients preferred the new generation of progressive lens (i.e., VARILUX PANAMIC ORMA CRIZAL; 50.0% of patients allocated to the new-generation lens first and 65.6% of patients allocated to the older-generation lens first), 36.5% preferred the old-generation lens (i.e., VARILUX CONFORT ORMA CRIZAL; 45.2% of patients allocated to the new-generation lens first and 28.1% of patients allocated to the older-generation lens first) and 5.6% had no preference (4.8% of patients allocated to the new-generation lens first and 6.2% of patients allocated to the older-generation lens first) (p = 0.01). Bifocal visual performance did not differ between the two groups, except for near visual acuity, which was better with the new-generation lens (i.e., VARILUX PANAMIC ORMA CRIZAL) (table 2). Figure 2 presents the mean scores for bifocal visual performance in periods 1 and 2. Table 3 describes the rapidity of visual adaptation with the older- and new-generation of lenses. The adaptation was faster with the new-generation lens (i.e., VARILUX PANAMIC ORMA CRIZAL).

**Table 2:** Secondary outcomes

**Bifocal visual performance, 0–10 scale**	**Period 1** N = 63 Mean (SD)	**Period 2** N = 64 Mean (SD)	P value*
**Near visual acuity**			0.02
-New-generation lens first	8.1 (2.3)	7.8 (2.2)	
-Older-generation lens first	7.9 (1.9)	8.5 (1.6)	
**Distance visual acuity**			0.38
-New-generation lens first	8.3 (2.0)	8.2 (1.7)	
-Older-generation lens first	8.2 (1.6)	8.4 (1.8)	
**Intermediate visual acuity**			0.46
-New-generation lens first	7.4 (2.6)	7.4 (2.3)	
-Older-generation lens first	7.7 (2.0)	8.1 (2.2)	
**Global visual acuity**			0.35
-New-generation lens first	7.7 (2.0)	7.6 (2.0)	
-Older-generation lens first	7.9 (1.5)	8.1 (2.1)	
**Distance visual field**			0.60
-New-generation lens first	7.6 (2.2)	7.7 (2.1)	
-Older-generation lens first	7.8 (1.6)	8.1 (1.9)	
**Near visual field**			0.16
-New-generation lens first	7.3 (2.4)	7.2 (2.2)	
-Older-generation lens first	7.0 (2.0)	7.7 (2.1)	
**Kinetic visual skills when the person is moving but the environment is still**			0.20
-New-generation lens first	7.5 (2.2)	7.5 (2.2)	
-Older-generation lens first	7.5 (1.8)	8.1 (2.0)	
**Kinetic visual skills when the person is still but the environment is moving**			0.22
-New-generation lens first	7.5 (2.2)	7.6 (2.1)	
-Older-generation lens first	7.7 (1.7)	8.2 (1.8)	
**Visual adaptability**			0.25
-New-generation lens first	7.6 (2.8)	7.7 (2.3)	
-Older-generation lens first	7.5 (1.9)	8.2 (2.2)	
**Visual comfort**			0.69
-New-generation lens first	7.6 (2.4)	7.6 (2.1)	
-Older-generation lens first	7.8 (1.5)	8.0 (2.4)	

* Study group effect tested by F test in the framework of a linear mixed-effect model

**Table 3:** Rapidity of adaptation to progressive lenses in periods 1 and 2.

Sequence	**Faster adaptation for the lens ** **of period 1 (immediate or few hours)**	**No difference**	**Faster adaptation for the lens of ** **period 2 (immediate or few hours)**	**TOTAL**
	**N**	**N**	**N**	**N**
New-generation lens then older-generation lens	14	35	12	61
Older-generation lens then new-generation lens	5	39	19	63

**TOTAL**	19	74	31	124

Patients had fast adaptation if they answered that the adaptation was immediate or in a few hours. Patients were considered as having a long adaptation if they answered that the adaptation was more than few hours.

Prescott 2-tailed test: p = 0.0315

We did not detect any statistically significant carry-over effect for secondary outcomes.

### Harm

No adverse events were reported during the study.

## Discussion

We compared the effectiveness of two progressive lenses prescribed for presbyopia in terms of patient preference for a lens and subjective measures of bifocal visual performance. To our knowledge, no study has rigorously assessed the effectiveness of the new generation of progressive lens (VARILUX PANAMIC ORMA CRIZAL) versus the older-generation lens (VARILUX CONFORT ORMA CRIZAL). For the primary outcome, approximately half of the patients preferred the new-generation lens and about one third preferred the older-generation lens. Although the period of wear seemed to influence patients' preference (i.e., patients' preference for the new-generation lens tended to be more pronounced when patients wore the old generation first), the effect was not statistically significant. The two groups did not differ in most of the secondary outcomes assessed (e.g., distance, intermediate, and global visual acuity; distance and near visual field; kinetic visual skills; visual comfort; visual adaptability) but did differ in near visual acuity, which was better with the new-generation lens. However, the difference was small (0.6 on a 0–10 numeric scale) and the clinical relevance of this difference is probably questionable.

Nijkamp et al. previously showed that quality of corrected near vision acuity was a predictor of patient satisfaction [14]. As well, lenses must fit properly. Adaptation to the lenses and comfort are important expectations for patients wearing lenses [15]. We found a tendency, although not significant, for an effect of period of wear, as shown in figure 2. In fact at the end of period 1, subjective assessment of the bifocal visual performance of lenses was similar for both groups, but subjective assessment was higher for wearers of the new-generation lens in period 2. Further, adaptation was significantly faster for the new-generation lens than with the older-generation lens.

Assessment of progressive lenses in presbyopia is a challenge because presbyopia is frequent and is related to elevated medical cost. In the past 4 years, only two randomised controlled trials have assessed interventions for presbyopia [14,16]. Methodological difficulties may explain this insufficiency in assessing interventions for presbyopia. Assessment of nonpharmacological interventions such as progressive lenses in presbyopia is difficult. Respect for scientific standards is important to guarantee the quality of assessment. The planning and conduct of this trial followed the guidelines established for nonpharmacological treatments [12,13,17]., but we encountered methodological difficulties. First, the choice of primary outcome was not easy. The primary outcome was a patient-reported outcome. We chose patient preference because comfort is of high importance for patients wearing progressive lenses. Second, blinding of patients was particularly important because outcomes were subjective and the outcome assessor was the patient. To guarantee the success of blinding, progressive lenses allocated were strictly similar but were all engraved with a tiny mark distinguishing the new versus older lenses. Although patients were not informed of this distinction, the presence of this mark could be considered a limit to adequate blinding.

## Conclusion

Patients showed a tendency to prefer the new generation of progressive lens for presbyopia. Approximately half the patients preferred the new-generation lens (i.e., VARILUX PANAMIC ORMA CRIZAL) and only one-third the older-generation lens (i.e., VARILUX CONFORT ORMA CRIZAL).

## Competing interests

This trial was funded by the Fédération Française des Sociétés d'Assurance – Direction Santé – 26, Bd Haussmann – 75009 Paris.

AZUR GMF – 8, rue Boissy d'Anglas – 75008 Paris

AVIVA – 3, rue du Moulin Baily – 92270 Bois Colombes

GENERALI – 7, Bd Haussman – 75009 Paris

GAN-GROUPAMA – 8–10 rue d'Astorg – 75008 Paris

SANTÉCLAIR – 78, Bd de la République 92100 Boulogne

## Authors' contributions

Conception, design, acquisition of data: IB, CT, PR. Analysis and interpretation of data: IB, CT, IP, CR, PR. Drafting the manuscript or revising it critically for important intellectual content: IB, CT, IP, CR, PR.

## References

[B1] Hermans EA, Dubbelman M, Heijde GL van der, Heethaar RM (2008). Change in the accommodative force on the lens of the human eye with age. Vision Res.

[B2] Weale RA (2003). Epidemiology of refractive errors and presbyopia. Surv Ophthalmol.

[B3] Nirmalan PK, Krishnaiah S, Shamanna BR, Rao GN, Thomas R (2006). A population-based assessment of presbyopia in the state of Andhra Pradesh, south India: the Andhra Pradesh Eye Disease Study. Invest Ophthalmol Vis Sci.

[B4] Ramke J, du Toit R, Palagyi A, Brian G, Naduvilath T (2007). Correction of refractive error and presbyopia in Timor-Leste. Br J Ophthalmol.

[B5] Luo BP, Brown GC, Luo SC, Brown MM (2008). The Quality of Life Associated with Presbyopia. Am J Ophthalmol.

[B6] Callina T, Reynolds TP (2006). Traditional methods for the treatment of presbyopia: spectacles, contact lenses, bifocal contact lenses. Ophthalmol Clin North Am.

[B7] Hersh PS (2005). Optics of conductive keratoplasty: implications for presbyopia management. Trans Am Ophthalmol Soc.

[B8] Meister DJ, Fisher SW (2008). Progress in the spectacle correction of presbyopia. Part 1: Design and development of progressive lenses. Clin Exp Optom.

[B9] Meister DJ, Fisher SW (2008). Progress in the spectacle correction of presbyopia. Part 2: Modern progressive lens technologies. Clin Exp Optom.

[B10] Sibbald B, Roberts C (1998). Understanding controlled trials. Crossover trials. Bmj.

[B11] Altman DG, Schulz KF, Moher D, Egger M, Davidoff F, Elbourne D, Gotzsche PC, Lang T (2001). The revised CONSORT statement for reporting randomized trials: explanation and elaboration. Ann Intern Med.

[B12] Boutron I, Moher D, Altman DG, Schulz KF, Ravaud P (2008). Extending the CONSORT statement to randomized trials of nonpharmacologic treatment: explanation and elaboration. Ann Intern Med.

[B13] Boutron I, Moher D, Altman DG, Schulz KF, Ravaud P (2008). Methods and processes of the CONSORT Group: example of an extension for trials assessing nonpharmacologic treatments. Ann Intern Med.

[B14] Nijkamp MD, Dolders MG, de Brabander J, Borne B van den, Hendrikse F, Nuijts RM (2004). Effectiveness of multifocal intraocular lenses to correct presbyopia after cataract surgery: a randomized controlled trial. Ophthalmology.

[B15] du Toit R (2006). How to prescribe spectacles for presbyopia. Community Eye Health.

[B16] Richdale K, Mitchell GL, Zadnik K (2006). Comparison of multifocal and monovision soft contact lens corrections in patients with low-astigmatic presbyopia. Optom Vis Sci.

[B17] Boutron I, Moher D, Tugwell P, Giraudeau B, Poiraudeau S, Nizard R, Ravaud P (2005). A checklist to evaluate a report of a nonpharmacological trial (CLEAR NPT) was developed using consensus. J Clin Epidemiol.

